# Structural robustness of mammalian transcription factor networks reveals plasticity across development

**DOI:** 10.1038/s41598-018-32020-1

**Published:** 2018-09-17

**Authors:** J. L. Caldu-Primo, E. R. Alvarez-Buylla, J. Davila-Velderrain

**Affiliations:** 1 Centro de Ciencias de la Complejidad (C3), Universidad Nacional Autónoma de México, Cd. Universitaria, México, D.F. 04510 Mexico; 2 Instituto de Ecología, Universidad Nacional Autónoma de México, Cd. Universitaria, México, D.F. 04510 Mexico; 3 MIT Computer Science and Artificial Intelligence Laboratory, Cambridge, Massachusetts USA; 4 Present Address: Broad Institute of MIT and Harvard, Cambridge, Massachusetts USA

## Abstract

Network biology aims to understand cell behavior through the analysis of underlying complex biomolecular networks. Inference of condition-specific interaction networks from epigenomic data enables the characterization of the structural plasticity that regulatory networks can acquire in different tissues of the same organism. From this perspective, uncovering specific patterns of variation by comparing network structure among tissues could provide insights into systems-level mechanisms underlying cell behavior. Following this idea, here we propose an empirical framework to analyze mammalian tissue-specific networks, focusing on characterizing and contrasting their structure and behavior in response to perturbations. We structurally represent the state of the cell/tissue by condition specific transcription factor networks generated using DNase-seq chromatin accessibility data, and we profile their systems behavior in terms of the structural robustness against random and directed perturbations. Using this framework, we unveil the structural heterogeneity existing among tissues at different levels of differentiation. We uncover a novel and conserved systems property of regulatory networks underlying embryonic stem cells (ESCs): in contrast to terminally differentiated tissues, the promiscuous regulatory connectivity of ESCs produces a globally homogeneous network resulting in increased structural robustness. We show that this property is associated with a more permissive, less restrictive chromatin accesibility state in ESCs. Possible biological consequences of this property are discussed.

## Introduction

A central tenet of systems biology is that cell behavior can be understood in terms of the structure and dynamics of underlying complex molecular networks^1,2^. Under such paradigm, major efforts have been made to systematically map and characterize the properties of molecular networks at different levels of organization. Reference protein-protein interaction, metabolic, and transcriptional regulatory networks have been constructed and are being frequently updated in several model organisms^3–5^. Initial efforts have largely focused on providing an organismal reference for the global network structure.

Network theory provides methods for the systemic description of a network’s structure and its dynamics^6–8^. One of the major results of network biology is the discovery within the reference networks of apparently universal organizational properties across the different types of complex biological networks^2^. While the characterization of reference real-world complex networks has uncovered structural similarities among complex networks that are believed to underly their systemic properties^2,6^, much less is known about the degree of structural heterogeneity of condition-specific biomolecular networks, and how patterns of variation promote or constrain systems-level behaviors.

In cell biology, one intriguing hypothesis is that network heterogeneity emanating from the normal process of development might result in differential behaviors underlying the contrasting cellular phenotypes. In line with this idea, the field of network biology has recently started shifting towards the characterization of condition-specific networks and analysis of circuitry dynamics^9,10^, presumably due to the increasing availability of functional genomics and epigenomics assays. For example, Neph and collaborators put forward a methodology to assemble tissue-specific transcription factor networks with the aid of available chromatin accessibility profiles from multicellular genomes^9,11–13^. The proposed networks connect each transcription factor (TF) to its incoming TF regulators, thus representing the regulatory structure of the cell in terms of the main regulators (e.g. TFs) and the mutual regulatory interactions among them. More specifically, using digital genomic footprinting (DGF) analysis, TF-TF interactions are established by integrating TF motif matching with DNase I hypersensitive sites (DHS) and high-resolution genomic footprints. Tissue-specificity comes from the condition-specific accessibility of cis-regulatory regions upstream a TF. Using this approach, tissue-specific TF networks have been constructed for model organisms and for human^9,14^. Given that the observed TF interactions reflect tissue-specific activity states, we reasoned that the structure and relative systems-level behavior displayed by these networks could provide insights into the biology and differentiation potential of the corresponding tissues.

In order to begin understanding the link between network structure heterogeneity, behavior, and biological phenotypes, here we put forward a computational framework to characterize the structural properties of mammalian tissue-specific TF networks and their behavior, emphasizing the degree of deviation from theoretical expectations. We focus on one systems-level behavior which is informative of the latter: the robustness of the networks to structural perturbations. We profiled the structural properties of a broad set of TF networks in mouse and human, and we compared the observed behavior across tissues and against expectations of theoretical models. Interestingly, we discovered that embryonic stem cells (ESCs) posses a distinctive regulatory structure: its higher structural similarity to the topological properties expected from a homogeneous network theoretical model endows them with a remarkable resilient behavior. We show by analysing chromatin accesibility profiles, that the tissue-specific TF network captures at a systems level, the more permissive and less restrictive property of the ESC epigenome relative to adult, differentiated tissues. However, unlike previous studies quantifying developmental potential with a gene expression-based network entropy framework^15,16^, we did not find a robust distinction between adult stem and differentiated cell populations; which might indicate a limitation of the degree of resolution captured by TF networks and, consequently, of the structural robustness measure proposed here. We discuss potential biological implications, and future extensions.

## Results

### Analysis framework

**Figure 1:**
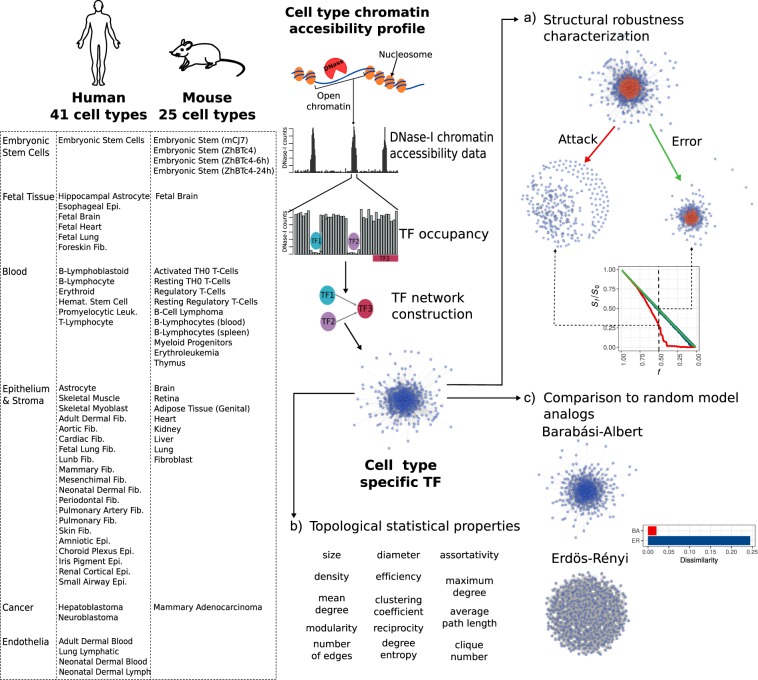
Structural profiling of cell type specific TF networks. (**a**) Structural robustness was measured simulating attacks (removing high degree nodes (red)) and errors (removing randomly selected nodes). (**b**) Networks characterization was done measuring topological features of every network. (**c**) Each network was compared to random model networks by measuring its dissimilarity to an analogous ensemble of homogeneous and scale-free networks.

Networks provide a theoretical framework that allows a convenient conceptual representation of interrelations among a large number of elements^6^. Furthermore, it is usually possible to frame questions about the behavior of the underlying real system by applying well-established analyses on the network representing empirical data^17^. Here we focus on tissue-specific networks where nodes represent TFs and links inter-regulatory interactions, and propose an analysis framework with the goal of characterizing the commonalities and differences in behavior against structural perturbations across tissues. We ask whether some tissues display extreme behaviors, and whether or not such deviations and extreme behaviors highlight aspects of the underlying biology. We hypothesize that the differences to be discovered underlie aspects of the observed biological functionality and of the broad degree of differentiation of the tissues. The proposed framework includes the following steps (see Fig. 1). (1) The state of the cell is structurally represented by tissue-specific networks of regulatory interactions among transcription factors as proposed in^9,14^. Briefly, a TF is considered regulator of another TF when a motif instance of the former TF occurs within a DNase I footprint contained in the proximal regulatory region of the latter TF (10 kb interval centered on the transcription start site [TSS]). (2) The system’s behavior of a network is defined as the response of the network against increasing structural perturbations^2,18^, and the response is measured by two metrics: the change in giant component size, and the change in efficiency, both relative to the original, unperturbed network (see Methods). The complete behavior is captured by the qualitative properties of the change from start until complete disruption; we introduce a simple metric to quantify it (Fig. 1a). (3) The structure of each network is numerically characterized by 14 topological measures (Fig. 1b). (4) The degree of deviation of each network relative to expectations from homogeneous (Erdős-Rényi) and heterogeneous (Barábasi-Albert) random graph models is quantified (Fig. 1c).

After applying these steps to each tissue-specific network, we rank the networks based on the robustness of their behavior, we identify those displaying the most extreme response, and we statistically explain the behavior in terms of predictive topological features and relative deviation from analogous homogeneous and heterogeneous random models. Thus, starting from an input set of tissue-specific networks, our framework produces a structural robustness ranking, a set of structural features underlying the behavior, and a mapping of the networks into the homogeneous-heterogeneous network space.

### Network structural differences reveal plasticity of systems behavior upon perturbation

It has been shown that a differential response to random structural perturbations (errors) and directed alterations (attacks) enables a concrete distinction between homogeneous and heterogeneous networks in terms of systems’ behavior^18^. A network representing a real complex system is expected to tolerate random failures, but to be more vulnerable against directed attacks targeting key, connected components. Taking this well-established framework, we evaluated the robustness behavior of TF networks across tissues. The operational definition of structural robustness applied here is based on an intuitive idea: disabling a substantial number of nodes will result in an inevitable functional disintegration of a network^2^, but the degree of tolerance will vary across tissues. We measured tolerance to random perturbations by randomly removing nodes from the networks and quantifying the change in the size of the largest connected component (giant component), and the change in network efficiency – an approximation to loss or gain of network connectivity (see Methods). For directed attacks, we repeated the experiments but sequentially removing nodes in decreasing order of centrality (degree) (Fig. 1a). We profiled the response to perturbations in 41 human and 25 mouse tissue networks.

**Figure 2:**
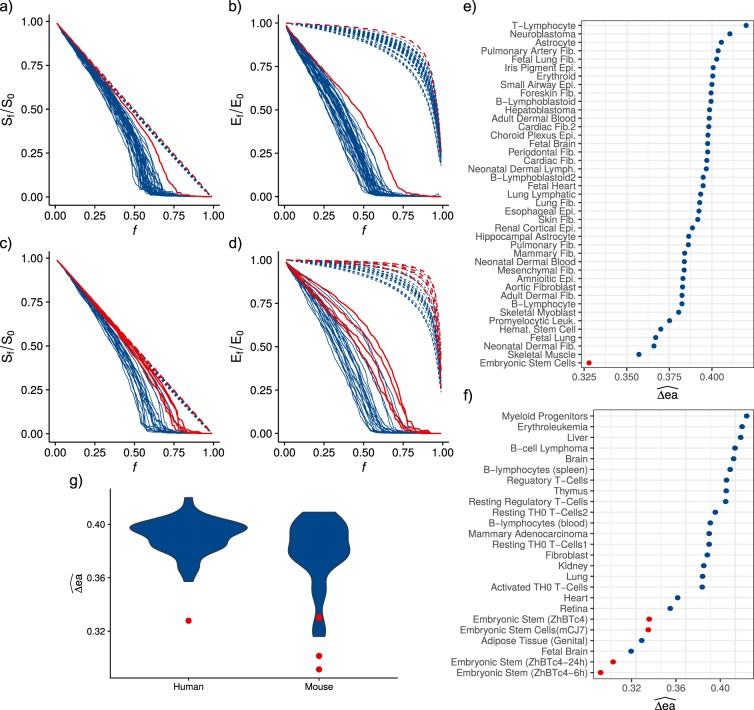
TF networks structural robustness. The behavior against errors (dashed) and attacks (solid) of every cell type is shown, red lines correspond to the ESCs behavior and blue lines to other cell types. (**a**) Human giant component size decrease. (**b**) Human efficiency decrease. (**c**) Mouse giant component size decrease. (**d**) Mouse efficiency decrease. (**e**) Human and (**f**) mouse vulnerability measure for each TF network. (**g**) Human and mouse vulnerability measures distribution, red dots correspond to ESC measurements.

Overall, all networks were found to be highly tolerant to random errors. In both mouse and human tissues, the size of the giant component (*S*_*f*_/*S*_0_) decreases linearly with *f* without abrupt transitions (Fig. 2a,c, dashed lines). The efficiency of the networks (*E*_*f*_/*E*_0_) also shows consistent behavior across all human and mouse tissues: it shows minimal decrease for a large proportion of *f* until it falls abruptly around *f* = 0.8 (Fig. 2b,d, dashed lines). The observed robustness to random failures is consistent with predictions from percolation theory in complex random networks, as it is less likely to perturb key, highly connected components in networks with long-tail degree distribution^6,18^. Also consistent with theory, TF networks were found to be much more vulnerable to directed attacks. Interestingly, however, we observed a high degree of variability in the behavior upon attacks across networks. Both measures (giant component size and efficiency) revealed transitions at different fractions *f* of attacked nodes (see Fig. 2a–d, solid lines). Interestingly, we found that in both human and mouse the TF networks of embryonic stem cells (ESCs) display, relative to differentiated tissues, an extremely robust behavior against both failure and attack perturbations, the latter being much more pronounced (see Fig. 2a–d, red lines).

With the goal of quantitatively describing and to analyze the discovered patterns of heterogeneity among tissues, we define the metric error-attack deviation (Δ*ea*), which simply quantifies the degree of deviation of a given network’s behavior upon directed attack perturbations from that stemming from random errors. We use this metric here as a measure of the structural robustness of complex networks to perturbations, as it reflects the degree to which attacks and errors are tolerated (see Methods). Intuitively, the smaller the value of Δ*ea* the closer the global response of the network against attacks relative to that against error, indicating a higher degree of robustness. We performed the calculation individually for the two damage measures used in this study: *S*_*f*_/*S*_0_ and *E*_*f*_/*E*_0_ (Supplementary Fig. 1). From these error-attack deviation measures, we defined network structural vulnerability ($\widehat{{\rm{\Delta }}ea}$) as the mean Δ*ea* for giant component size and efficiency (see Methods). This measure enables the quantification of differential structural robustness to attacks displayed by the networks (cell types). The vulnerability measure of human and mouse cell types corroborates the heterogeneity of structural robustness among cell types, and the extremely deviating behavior of ESCs (Fig. 2). ESCs have an error-attack deviation significantly lower than other cell types, highlighting their significantly higher robustness against attacks relative to more differentiated tissues.

### Network structural rearrangement during differentiation

The observed differences in structural robustness among tissues point to the existence of patterns of variation in global network structure. In order to characterize the structural heterogeneity of TF networks, we analyzed their topology and asked whether specific topological features more predominantly explain the observed robustness patterns. In particular, what structural features underlie the extreme robust behavior of ESCs? As a first approximation we simply asked how similar are networks among each other? We computed pair-wise dissimilarity scores for every pair of TF networks in mouse and human, using a structural dissimilarity (*D*) approach (see Methods). Network dissimilarity is a useful method for network comparison as it quantifies structural topological differences based on node distance probability distributions, capturing nontrivial structural differences^19^ – as opposed to the intuitive counting of presence or absence of common links.

**Figure 3:**
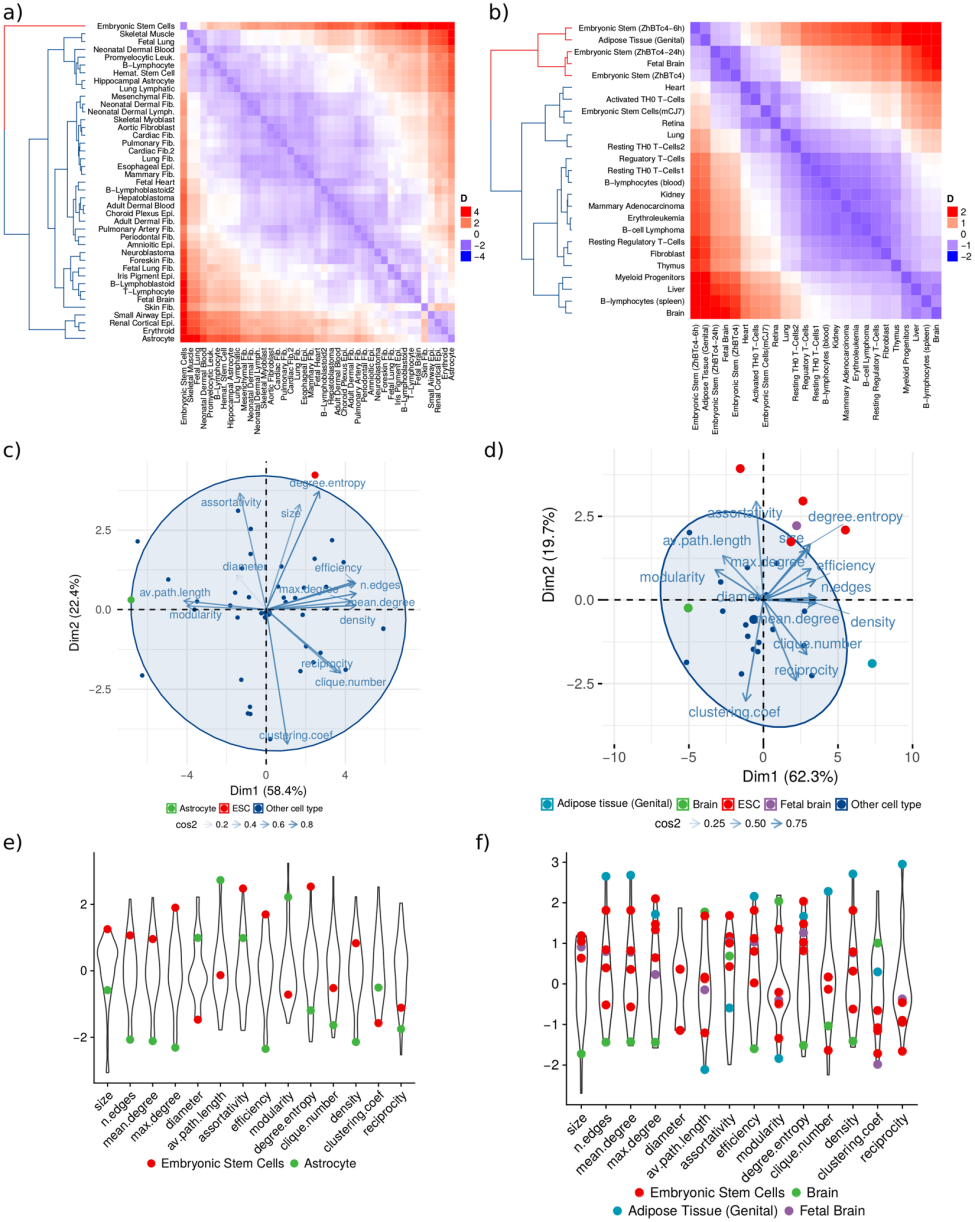
Networks structural profiling. (**a**,**b**) Dissimilarity among cell types, heatmaps of scaled *D* values among (**a**) human and (**b**) mouse cell types. (**c**) Human and (**d**) mouse networks topological features PCA. (**e**) Human and (**f**) mouse topological features distribution, colored dots show the value for each feature of the indicated cell type.

Despite the fact that all TF networks are relatively similar – having average *D* values of 0.040 and 0.064 in human and mouse, respectively – there is variation in the structural similarity among them. *D* ranges from 0.003 to 0.160 in human, and from 0.003 to 0.184 in mouse. Considering pair-wise comparisons in human networks, ESC is the most dissimilar network for 24 (58.5%) of the tissues. For the remaining 17 tissues, the most dissimilar network corresponds to Astrocyte. These two tissues also have the highest *D* median scores: ESC (0.090) and Astrocyte (0.077). Interestingly, these two networks are also the most dissimilar between one another. Thus, the undifferentiated ESC localizes at one extreme of the topological space while the highly differentiated Astrocyte localizes at the other. We built a dendrogram using network dissimilarity as distance measure among human networks. ESC is clearly different from the other tissues as it is placed in a single branch at the bottom of the distance dendrogram, separated from all the other cell types (Fig. 3a).

Mouse networks show a similar pattern to that found in human cell types. Pair-wise comparisons show that the most dissimilar networks are ESCs and the highly differentiated Brain, with these two tissues occupying the extremes in the dissimilarity distribution (Fig. 3b). ESC ZhBTc-6h has the highest *D* value for 16 of the 25 cell types (64%), while the other two ZhBTc ESCs also rank among the most different networks, and in the remaining 9 cell types the highest *D* value corresponds to Brain. Unbiased hierarchical clustering aggregates three ESC lines (ZhBTc, ZhBTc-6h, and ZhBTc-24h) in a separate basal branch, together with Genital Adipose Tissue and Fetal Brain. Fetal tissues are expected to display some degree of similarity with ESCs, due to overlap of developmental processes during fetal development. Adipose is an heterogeneous tissue, possibly including undifferentiated adipose stem cells. Overall, the topology of ESCs networks in mouse and human is clearly distinct from adult differentiated tissues such as brain and liver. In both dendrograms, differentiated tissues do not seem to be structured according to their lineage. This reflects that from a structural point of view, developmental lineages networks are not clearly distinguished and only a significant difference between ESCs and adult differentiated tissues is observed (Fig. 3). We reasoned that this observation might stem from a distinctive chromatin accesibility state charcaterizing ESCs, which we explore below. Overall, there is a significantly higher dissimilarity between ESCs and adult cell types than among those differentiated tissues (Supplementary Fig. 2).

To further explore the topological differences among tissues, we characterized the structure of every network using 14 standard measures for network topology description (Table 1, see Methods)^6,7^. These measures capture important characteristics of a network’s global structure, which in part determines its functionality. In particular, we seek to dissect the structural heterogeneity among tissues, identify features associated with the observed robustness, and finally map those structural features that discriminate ESCs’ networks from those of differentiated tissues.

**Table 1:** Topological features measured for every human and mouse network.

Feature	Human			Mouse		
	Range	Average	Standard Deviation	Range	Average	Standard Deviation
Size	[493, 533]	521	9.27	[555, 583]	574.4	7.21
No. of edges	[9099, 18906]	14002.97	2272.36	[15392, 36448]	22970.4	5084.83
Mean degree	[36.03, 72.02]	53.65	8.33	[54.10, 125.036]	79.816	16.881
Diameter	[5, 8]	6.19	0.81	[5, 7]	5.76	0.66
Density	[0.034, 0.069]	0.052	0.008	[0.48, 0.10]	0.07	0.014
Average path length	[2.30, 2.69]	2.45	0.086	[2.08, 2.41]	2.26	0.086
Clique number	[14, 27]	19.39	2.68	[19, 34]	26.44	3.31
Clustering Coefficient	[0.24, 0.39]	0.29	0.036	[0.25, 0.37]	0.30	0.027
Assortativity	[−0.21, −0.12]	−0.17	0.022	[−0.18, −0.13]	−0.15	0.015
Efficiency	[0.47, 0.54]	0.51	0.017	[0.50, 0.59]	0.54	0.024
Modularity	[0.10, 0.17]	0.12	0.014	[0.06, 0.11]	0.09	0.013
Degree Entropy	[5.69, 5.94]	5.80	0.052	[5.79, 6.09]	5.93	0.078
Reciprocity	[0.03, 0.06]	0.05	0.006	[0.04, 0.09]	0.06	0.01
Maximum degree	[266, 416]	348.2	35.6	[334, 574]	436.9	65.2

We performed principal components analysis (PCA) using the measured topological features, in order to explore network aggregation behavior in the feature space, while at the same time avoiding collinearity. For both human and mouse data, the features with highest contribution for the first principal component (PC) are mean degree, number of edges, density, efficiency, and modularity. The former four features are highly correlated, all of them measuring network degree of connectivity. In spite of mean degree’s high contribution to the first PC, this property does not explain the structural difference observed in ESCs: mean degree of ESCs does not deviate from the empirical distribution among other tissues (Supplementary Fig. 3, and Fig. 3e,f). The features contributing to the second PC are clustering coefficient, assortativity, and degree entropy. Projecting the networks to a 2D space based on PCs, we found no apparent clustering (Fig. 3c,d). However, a closer examination shows that, as expected, ESCs are separated from the other tissues, having higher values for the second PC. The highly specialized networks of Astrocyte and Brain tissue localize at the opposite extreme, evidencing the extreme structurally differences relative to ESCs. These differentiated networks are characterized for having extremely low values for the first PC. Considering these patterns, ESCs are characterized for having high values of degree entropy and assortativity, but low clustering coefficient. On the other hand, Brain and Astrocyte networks have high modularity and average path length, but small density, efficiency, and mean degree. This pattern is confirmed by the features distribution (Fig. 3e,f).

The topological characterization corroborates an extreme difference in network topology between undifferentiated ESCs and differentiated tissues. Analysis of features distribution shows that tissues spread through a feature space following two main axes, one going from highly modular to highly efficient networks, and another separating highly degree entropic and degree assortative structures from those with high global clustering. ESCs are distinguished from differentiated tissues for having more interacting TFs, and these are globally connected in a more promiscuous way, as evidenced by higher levels of entropy in the degree distribution. In contrast, differentiated networks of Brain and Astrocyte are more structured, as evidenced by high levels of modularity, yet low levels of efficiency and density. Taking into account the existence of a trade-off between network efficiency and modularity^20^, this observation hints to a possible path of developmental dynamics of TF network structure in which the system transits from a configuration promoting efficiency in information flow and robustness, into a highly modular topology suggestive of functional specialization.

### Interpretation in terms of theoretical network models

As mentioned above, robustness to directed attacks has been linked to homogeneous network topologies, in contrast to the “robust yet fragile” behavior characteristic of heterogeneous (scale-free) networks^18^. Considering this result, we compared each TF network to analogous ensembles of random homogeneous and scale-free networks generated using the Erdős-Rényi (ER) and the Barabási-Albert (BA) models, respectively (see Methods). ER networks with high number of nodes approach a Poisson degree distribution, symmetric for relatively high average degrees. On the contrary, BA networks have a characteristic right skewed power-law degree distribution. We compared the real world networks with the theoretical models, with the goal of placing them within a heterogeneity axis by quantifying deviations. Given the discovered high robustness to directed attacks and high degree entropy of ESCs, we reasoned that such a contrast will help clarify the global structural features underlying such behavior.

**Figure 4:**
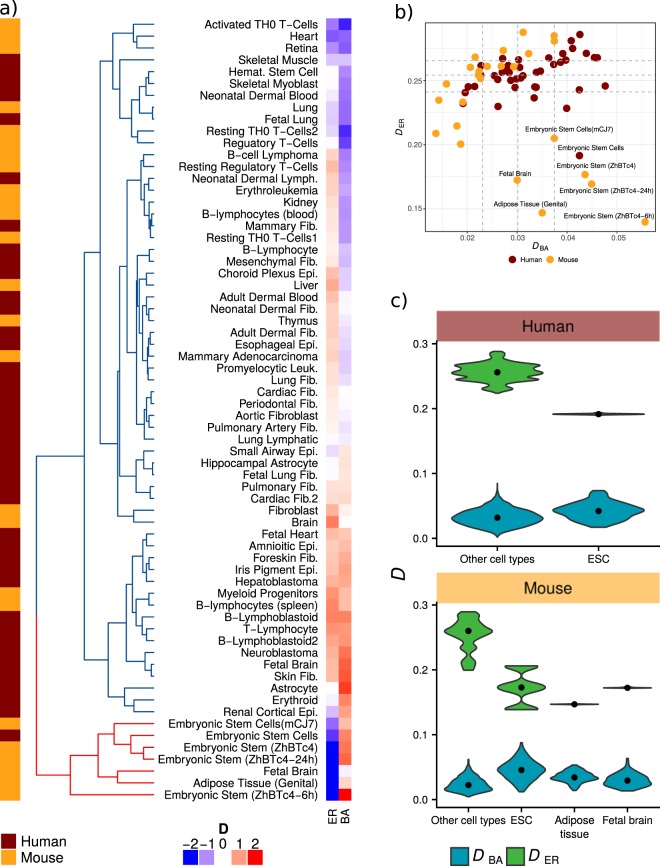
Networks comparison with ER and BA model networks. (**a**) Heatmap human and mouse cell types dissimilarity to model networks. (**b**) Scatterplot of cell types dissimilarity to model networks, dashed lines in both axis correspond to the 25, 50 and 75 percentiles of both measurements. (**c**) Distribution of *D* values for ESCs and other cell types in human and mouse, respectively. Distributions correspond to the dissimilarity with each of the 100 simulated model networks, black dots correspond to distribution median.

We measured network structural dissimilarity between each network and its ER (*D*_*ER*_) and BA equivalents (*D*_*BA*_). As expected, all networks are significantly more similar to BA than to ER networks. *D*_*ER*_ ranges from 0.191 to 0.285 and from 0.139 to 0.287; whereas *D*_*BA*_ ranges from 0.019 to 0.047 and from 0.013 to 0.055, in human and mouse respectively (Fig. 4c). The fact that BA networks are more similar to the TF networks is consistent with discoveries of other real world complex networks having scale-free topologies^21^. Interestingly, however, we found clear differences among the networks regarding their relative similarity to each theoretical model. For instance, ESCs have the lowest *D*_*ER*_ in both human and mouse (Fig. 4). In the case of *D*_*BA*_, a contrasting pattern emerges: ESCs are among the tissues with higher values. Nevertheless, ESC *D*_*BA*_ values are not significantly different from those of other tissues, falling within the observed distribution of *D*_*BA*_ (Fig. 4c). Considering *D*_*ER*_ and *D*_*BA*_ together and taking both human and mouse networks, ESCs are separated from the other cell types, as shown in Fig. 4. A conserved pattern in both human and mouse emerges in which ESCs have a relatively lower dissimilarity to ER networks and a relatively higher dissimilarity to BA than the other tissues. As with the dendrogram created from *D* among networks (Fig. 3b), dissimilarity to model networks does not recover lineage hierarchies of differentiated tissues, yet it underscores a broad difference in global structure between ESCs and differentiated tissues.

**Figure 5:**
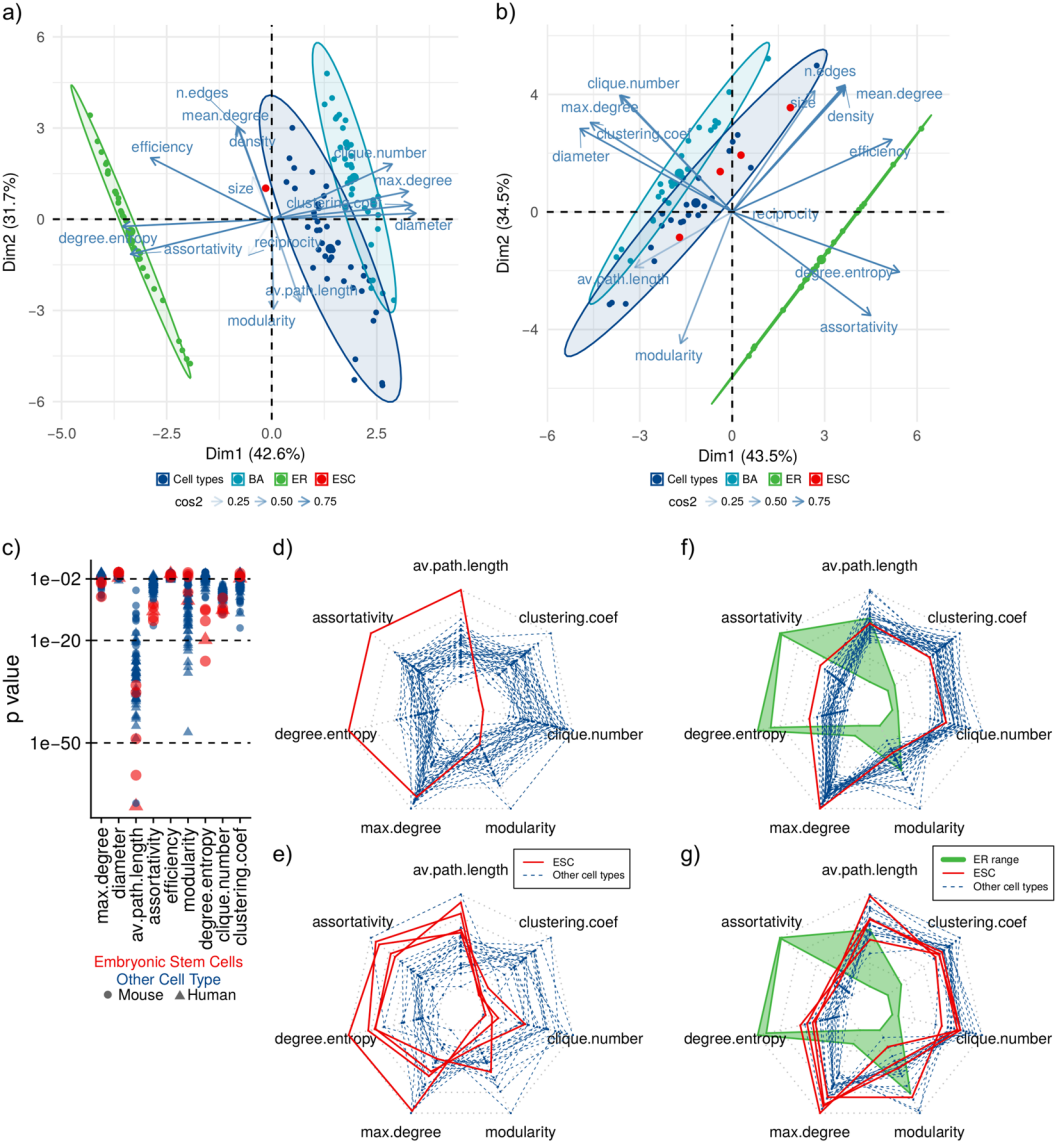
Comparison of network features with random model analogs. (**a**) Human and (**b**) mouse network features PCA including real, ER, and BA networks. (**c**) Network features p values comparing real features with BA model analogs. (**d**) Human and (**e**) mouse radar plots of network features z-score compared to BA model analogs. (**f**) Human and (**g**) mouse features measures, green polygons show ER networks’ range for each feature. ESC values are shown in red solid lines, and the other cell types are shown in blue dashed lines.

For every model network we measured the same 14 topological properties we used to characterize cell type networks, and performed a PCA of their features including the real and model networks. In both human and mouse networks, the PCA graph shows a common pattern. The first component separates three clusters corresponding to each model network and the real networks, situating BA and ER networks in the extremes and the real networks between them, closer to the BA cluster (Fig. 5a,b). As shown in the structural dissimilarity analysis, this pattern confirms that real networks are more similar to scale-free networks than homogeneous networks. Real TF networks are situated in between BA and ER clusters, thus creating a feature space between the two model networks in which real networks can be situated. The pattern shows that ER networks tend to have higher degree entropy and assortativity, while BA networks tend to have higher diameter and clustering coefficient (Fig. 5a,b).

We show that ESC networks have a distinctly higher robustness against directed attacks relative to differentiated tissues. Since scale-free topology explains the fragility against directed attacks in complex networks, we analyzed the topological features of ESCs networks that deviate from BA expectations. We calculated the deviation of every real network feature compared to its distribution in the corresponding BA model (see Methods). From this analysis we selected features in which the real networks differ significantly from BA models, these are: average path length, assortativity, degree entropy, maximum degree, modularity, clique number, and clustering coefficient (Fig. 5c). We found extremely high deviation (z-score) on degree entropy, assortativity, and average path length in ESCs (Fig. 5d). From our PCA analysis, we know that these features have a high contribution to the first PC; in particular, ER networks tend to have higher degree entropy and assortativity. This indicates that, even though ESC networks are closer to a BA topology, the features for which they are different from a BA model are characteristic of ER networks. This is illustrated by visual contrast of ER expected and empirically observed values of the deviating features among cell types (Fig. 5f,g). Thus, we conclude that ESCs have extreme values in features characteristic of ER networks.

### Network homogeneity predicts structural robustness

**Figure 6:**
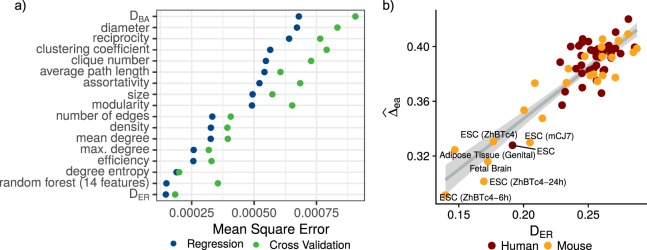
Predictive models. (**a**) Mean square error for linear regressions of network vulnerability using each feature as predictor and random forest using 14 topological features. (**b**) Linear regression of network vulnerability predicted by networks’ dissimilarity to Erdős-Rényi model network.

We show that the topological plasticity of tissue-specific TF networks can be characterized by comparing them to model networks. As mentioned before, this structural differences are associated with the networks’ response to random and directed perturbations. To further understand the structural features underlying the observed structural robustness pattern, we fitted statistical models in an attempt to further uncover explanatory topological features. Using the previously defined network vulnerability ($\widehat{{\rm{\Delta }}ea}$) as the response variable, we fitted two statistical models: linear regressions using the 14 network features as well as *D*_*ER*_ and *D*_*BA*_ as predictors, and a random forest regression using the 14 topological features as predictors. For each model we measured its mean square error, and validated its accuracy through five-fold cross validation. The best predictor of network vulnerability is network’s *D*_*ER*_ with a cross validation mean square error of 0.00022. There is a positive relationship between *D*_*ER*_ and $\widehat{{\rm{\Delta }}ea}$ (Fig. 6b), indicating that the more a network resembles a homogeneous network, the higher its structural robustness. The topological feature with the best predictive performance is degree entropy, a feature correlated with a network similarity to a homogeneous network. Thus, the diviation from ER model expectation *D*_*ER*_, a measure quantifying the degree of homogeneity of a real-world network, and which is distinctively high in ESCs; is predictive of structural robustness.

### ESCs TF network structure captures a more accessible and permissive chromatin state

Network structural analyses show that ESCs have a distinct network topology, mainly characterized by a higher uniformity in the number of interacting partners (degree entropy). Since the networks we analyzed reflect both the presence of a TF motif and DNase-seq chromatin accessibility signal^9,14^, we reasoned that at the global level, the distinctive network structure might capture an underlying, more permissive chromatin accessibility state, which has been previously hypothesized to underlie ESC behavior^14,22^. We tested this hypothesis empirically by directly analyzing DNase-seq chromatin accessibility data from the Roadmap Epigenomics project^23^, comparing samples corresponding to ESCs and adult differentiated cell types.

**Figure 7:**
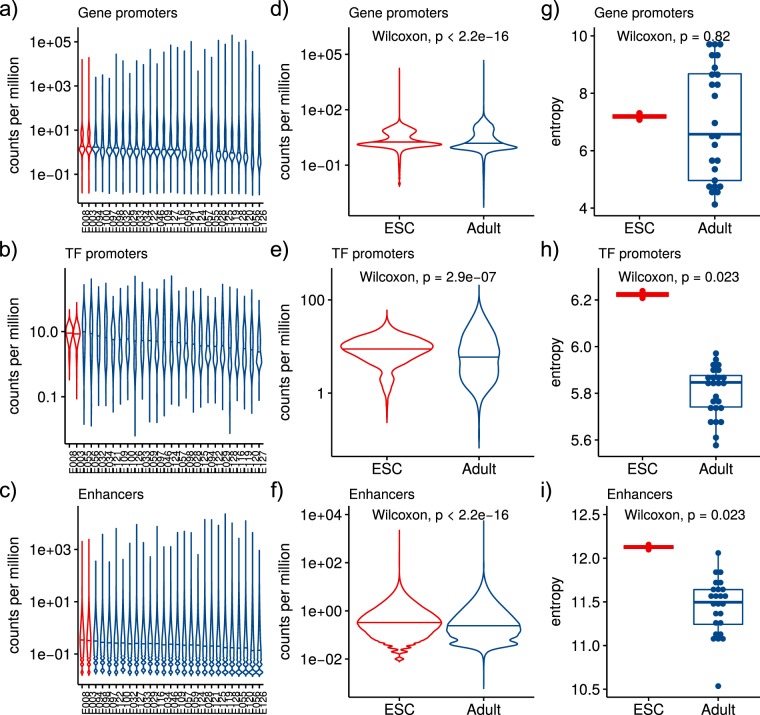
Chromatin accessibility in ESCs and adult tissue samples from Roadmap Epigenomics data^23^. Number of DNase-seq tags per million reads in (**a**) gene promoters, (**b**) TF promoters, and (**c**) enhancers for each sample. Group mean accessibility distribution in (**d**) gene promoters, (**e**) TF promoters, and (**f**) enhancers. Boxplot of groups accessibility distribution entropy in (**g**) gene promoters, (**h**) TF promoters, and (**i**) enhancers. Horizontal lines inside violin plots correspond to the distribution median.

We compared accessibility signal (normalized counts) across all gene promoters, TF promoters only, and enhancers, considering these entities key regulatory elements in transcriptional networks (REs). Overall, REs display higher median accessibility in ESC than in adult samples (Fig. 7a–c). To test group differences between ESCs and adult tissues, we defined for each regulatory region a mean accessibility score, and found that REs are significantly more accessible in ESCs than adult tissues in the three cases (Fig. 7d–f).

The accessibility distribution reveals a higher median accessibility in regulatory regions within ESCs and shorter tails in the extremes of the distribution, relative to adult samples. This points to a more evenly distributed activity among REs, a pattern particularly pronounced in TF promoters. To quantify this observation, we measured the entropy of the accessibility distribution. TF promoters and enhancers show a significantly higher entropy in ESCs compared to adult differentiated cell types (Wilcoxon, *p* =< 0.023) (Fig. 7h,i). This result indicates that, the main elements of the regulatory circuits specifying cell-identity (enhancers and TFs)^24^, display a distinctive, promiscuous activity (as approximated by accessibility) in ESCs. The reduction of uncertainty in RE activity observed in adult differentiated cell types evidences a more restrictive epigenomic state, in which some TFs and enhancers have high activity and influence on the identity of the cell state. On the other hand, the state of uncertainty in the accessibility of the REs resulting in permissive global activity of TFs and target REs in ESCs may be ultimately manifested in the pluripotent, undecided, and promiscuous nature characteristic of ESCs^22,25^. These contrasting permissive and restrictive patterns of accessibility, in particular in the neighborhood of TF TSSs, is captured in the network structures analyzed herein.

The more accessible, permissive, and promiscuous activity of regulatory elements and regulators (TFs) in ESC populations is consistent with both their pluripotent nature and with an increased robustness of the TF networks characterizing their state.

## Discussion

It has been pointed out that insights into the interplay between network structure and dynamics are needed in order to ultimately understand the cell’s functional organization^2^. Here we studied TF networks’ structure with the goal of better understanding the global behavior of different tissues. As a simple operational approximation, we represented the cell using tissue-specific TF networks. We frame the problem in terms of global structural robustness, a systemic behavior approximated by the vulnerability of networks to both random failure and directed perturbations^2,18^. We found that structural robustness varies significantly across tissues with different levels of differentiation. Interestingly, within the datasets analyzed in both human and mouse, the most robust tissue was also the least differentiated: embryonic stem cells.

Complex network theory has shown the coexistence of extremes in robustness and fragility (“robust yet fragile”) in real-world networks, due to the widespread power-law connectivity distribution associated with complex networks^18,26^. The networks underlying ESCs are the most robust against random failure as well as the least fragile against directed attacks, somehow being able to negotiate the observed trade-off between robustness and fragility. It is known that deviation from the long-tail of theoretical networks with power-law degree distribution reduces the effectiveness of an attack strategy based on targeting the highly connected nodes^18^. Although all the TF networks analyzed here do have a long-tailed degree distribution, they deviate from theoretical power-law degree distributions (see Supplementary Figs 4 and 5). We analyzed this deviation from a canonical scale-free network by measuring each network’s dissimilarity to theoretical model networks with homogeneous and scale-free topologies. This comparison further exposed the structural heterogeneity among tissues, and the deviating behavior of both undifferentiated (i.e., ESCs) and differentiated tissues. Furthermore, within the proposed analysis framework, the relative (dis)similarity between a target network and analogous theoretical networks provides insights into the topological characteristics underlying its robustness. For example, the higher structural robustness of ESC networks is explained by its closer topological resemblance to an Erdős-Rényi homogeneous random network, relative to differentiated cell types.

In terms of biological properties, our results suggest that ESC state might be able to withstand more and different kinds of errors, due to a more homogeneous network topology. This topological arrangement implies that its main regulator TFs act upon a less constrained chromatin landscape, allowing them to explore it more freely than in differentiated cell types. We further explored this idea by directly analyzing accessibility data at genome REs (TF promoters and enhancers), comparing ESCs and adult differentiated tissues. We found ESCs have a significantly higher accessibility at regulatory elements compared with differentiated tissues. ESCs also have a more evenly distributed accessibility among REs as shown by a higher entropy in its distribution (Fig. 7). Consistent with our results, several studies show that ESCs nuclear DNA is organized in an unusual way, in which chromatin appears to be more “open” than in differentiated cells^27^. Some of these findings are that histones and non-histones proteins are more loosely bound to DNA in ESC^28^, constitutive heterochromatin is more dispersed^28,29^, modifications associated with silent chromatin are depleted, while those associated with transcriptional activity are globally enriched^28,29^. These data has lead to consider stem and dedifferentiated cells as a state of loose regulation, differentiation being considered as a process of increasing chromatin repression^27,28,30^. Our results that show ESCs have a more homogeneous and structurally robust TF network topology can be considered a consequence of this loose regulation state in ESCs.

Previous studies have found a correlation between the level of uncertainty in the expression profile of a cell’s signaling network and its differentiation potential (pluripotency)^15,16^. In other words, pluripotent cells can be characterized by a state of high uncertainty, where molecules from opposite lineages are promiscuously and simultaneously expressed. This uncertain state seems to mechanistically promote a cell-fate decision, due to its instability^31,32^. Entropy-based measures of uncertainty have been shown to capture such degree of instability and therefore pluripotency: lineage committed cells would have reduced entropy relative to progenitors, as differentiation is associated with the predominant expression of one of the mutually competing transcriptional programs. Consistent with this view, a network entropy measure integrating tissue-specific transcriptomic profiles with a protein interaction network, has effectively quantified cellular pluripotency using bulk^15^ and single-cell data^16^.

In the present study we found that the structural robustness of a transcription factor network clearly discriminate ESCs from differentiated cell types. Unlike transcriptomic analyses, however, this property does not seem to correlate with cellular differentiation potential within specific lineages. One potential interpretation for this observation is that the analyzed networks may highlight differences in chromatin organization that might anticipate transcriptional differences between cell types. On the other hand, the inability of these measures to distinguish between multipotent and fully differentiated cell types could stem from a lack of resolution to capture more subtle differences in network arrangement, or from the loss of information during TF networks inference due to the averaging intrinsic to bulk DNA-seq data. Nonetheless, our results do highlight an association between pluripotency and uncertainty of the regulatory network state, as measured by the entropy of chromatin accessibility profiles. This observation is consistent with the general model of a molecularly promiscuous cellular state underlying pluripotency. Here uncertainty is measured from chromatin accessibility profiles, while previous, higher resolution studies used transcriptomic data^15,16^. An interesting research direction would be to study the precise relation between the two measures of entropy, linking epigenomic structural data with transcriptomic profiles. In particular the recent development of single-cell resolution chromatin accessibility^33^ and transcriptomic^34^ profiling technologies might enable disentangling associations between multiple levels of regulation, perhaps overcoming the limitations of inferring TF networks based on bulk data alone.

It is well know that network topology plays a central role in dynamical behavior. In the cellular context, gene regulatory networks orchestrate cellular behavior^35^. Theoretical studies have previously analyzed the interplay between structure and dynamics using random Boolean networks^36,37^. Networks with a homogeneous topology and relatively high connectivity require fine tuned activation parameters in order to have a stable behavior, and to avoid chaotic dynamics^36,37^. This result seems inconsistent with the nature of real biological systems, which have a stable behavior despite fluctuations in surrounding environmental parameters. In other words, resilience is a characteristic of biological systems. Interestingly, for networks with a scale-free topology stable behavior emerges without the fine tunning requirement^36,37^. Considering our results in this structure/dynamics context, the higher homogeneity found in the ESC networks is likely to produce less ordered dynamics than more differentiated tissues, which, at the same time would allow them to explore more freely the state space and to reach multiple different network states. Interestingly, this view is consistent with the observed high heterogeneity in gene expression and with the balance between robustness and plasticity characteristic of ESCs^15,25,38,39^. Although we did not consider dynamical analysis in this study, but rather limited ourselves to the empirical, structural characterization of the networks and their behavior, disentangling structure and dynamics will be the focus of future work.

Summarizing, in light of the amount of data on biological interactions being generated in the post-genomic era, a systems level perspective is required to gain understanding of the biological systems as a whole. Our structural analysis of tissue specific TF networks aims at that objective, trying to find a connection between transcriptional networks structural heterogeneity and biological phenotypes. Our treatment of structural robustness as a network systems-level behavior revealed differences among cell types that could be dissected further through topological analyses and related to chromatin accessibility profile at REs. We want to stress the applicability of our comparison of real world complex networks not only for a structural characterization, but also as an approximation to their possible dynamic behaviors. Finally, the empirical analysis framework proposed here can be applied to any set of related networks whose structural heterogeneity is suspected to underly differential real life behavior.

## Methods

### Transcription Factor Networks

Human and mouse transcription factor networks (TFNs) were constructed based on DNase-seq data and digital genomic footprinting as shown in^9,14^. Human networks set include 41 distinct cell and tissue specific networks composed of 493 to 533 sequence-specific transcription factors. Mouse networks set include 25 cell and tissue specific networks composed of 555 to 583 sequence-specific transcription factors. For simplicity, we use the term tissue-specific through the text to refer to both cell type and tissue. Network data were downloaded from https://www.regulatorynetworks.org/. Most current versions for human (v09162013) and mouse (v12032013) were used.

#### Modeling topological robustness

Topological robustness was approximated by profiling the network’s behavior in response to random and directed structural perturbations. Site percolation was used as a process to model component failure using computer simulations^6^. Increasing fractions of a network’s vertices were removed, along with the edges connected to those vertices. Following^6,40^ a percolation process was considered in the general sense – i.e., including different ways of vertex removal. The error experiments performed correspond to the simplest percolation process where a fraction of vertices was chosen uniformly at random and removed. For every network, error experiments were repeated 1000 times and the mean error behavior was calculated. Directed (Attack) experiments were simulated by removing vertices in decreasing order of centrality based on vertex degree. Nodes were progressively removed from one to a hundred percent of nodes.

### Quantifying network structural robustness

Two quantitative measures of network damage were used to characterize the phenomenology associated to the damage process applied to each TF network. As a first approximation, the macroscopic (systemic) behavior of the networks in response to damage was characterized by the evolution of the giant component size relative to its initial value as a function of the fraction of removed vertices *f* (*S*_*f*_/*S*_0_). As an additional approximation, the global efficiency *E* of a network was used to quantify how communication becomes less efficient as damage increases, this measure was also calculated relative to its initial value and as a function of the fraction of removed vertices *f*. The latter measure assumes that the efficiency for sending information between two vertices *i* and *j* is proportional to the reciprocal of their distance, and is calculated as follows^7,8^:

1 $$E=\frac{1}{N(N-1)}\,\sum _{i\ne j}\,\frac{1}{{d}_{ij}}.$$

The measure *E* corresponds to the average inverse geodesic length – i.e., the harmonic mean of the geodesic distances^7^:

2 $$h=\frac{1}{E}.$$

### Error-Attack Deviation and vulnerability calculation

The measure error-attack deviation Δ_*ea*_ introduced herein, was used to quantify the degree of robustness to attacks relative to that against errors. The metric is simply the root mean square deviation between the observed error and the attack behaviors:

3 $${{\rm{\Delta }}}_{ea}=\sqrt{\frac{1}{n}\,\sum _{f}\,{({e}_{f}-{a}_{f})}^{2}}$$

where *e*_*f*_ (*a*_*f*_) represents the a normalized measured of damage behavior under the random or (directed) removal of a fraction *f* of nodes. In this study *S*_*f*_/*S*_0_ and *E*_*f*_/*E*_0_ were used as damage measures (see Results).

We defined network vulnerability ($\widehat{{\rm{\Delta }}ea}$) as the mean between error-attack deviation to giant component size and efficiency:

4 $$\widehat{{\rm{\Delta }}ea}=\frac{{\rm{\Delta }}e{a}_{Sf/S0}+{\rm{\Delta }}e{a}_{Ef/E0}}{2}$$

#### Networks Topological Characterization

Networks’ topology was analyzed by quantifying topological dissimilarity and measuring 14 structural features commonly used in complex network theory^6,7^.

### Network dissimilarity

Network dissimilarity measurement was done following the approach proposed by Shieber *et al*.^19^. This method compares networks topology based on quantifying differences among node distance probability distributions, representing all nodes connectivity distances, extracted from the networks. It returns non-zero values only for non-isomorphic graphs, and quantifies structural topological differences that have an impact on information flow through the network. We measured network dissimilarity following the algorithm proposed in^19^, using the suggested parameters.

### Networks structural characterization

We described networks’ topology by measuring 14 features: number of nodes, number of edges, mean degree, diameter, maximum degree, average path length, density, clustering coefficient, assortativity, efficiency, modularity, degree entropy, clique number, and reciprocity. Following the measurement definitions in^7^.

### Null models

To compare cell type networks with random models, we generated random networks with the same number of nodes and links. Two sets of random networks were created: one set following Erdős-Rényi model (ER networks) with exponential degree distribution, and the second set following Barabási-Albert model of growing networks with power-law degree distribution (BA networks). In order for the BA networks to have an equivalent number of edges to its real counterpart, the number of outgoing edges added to each new node in the network was taken from the out degree distribution of the real network.

For each real network, 100 ER and BA random networks were created. Every random network was structurally characterized measuring the 14 topological features measured in the real networks, and dissimilarity to its real equivalent was quantified. Mean values for the dissimilarity and topological features were estimated for each ensemble of random networks.

### Features significance with respect to BA analogs

For each cell type, we constructed a feature BA analog expected distribution from the feature’s value in the 100 analog random BA networks. We then calculated the real feature z-score with respect to the BA expected distribution and using this z-score we obtained the p-value for each feature in every network.

#### Predictive modeling

Predictive models were fitted using networks’ vulnerability as a response variable and structural features as predictors.

First we fitted a linear regression predicting $\widehat{{\rm{\Delta }}ea}$ using the 14 statistical features we measures, plus the network’s dissimilarity to its ER analogs (*D*_*ER*_) and to its BA analogs (*D*_*BA*_) as predictors. The second model we fitted was a random forest regression, predicting $\widehat{{\rm{\Delta }}ea}$ from the 14 topological features measured above, this model was was created with 1000 trees. Features’ influence on the random forest model was measured by the mean decrease in mean square error. As a way to evaluate the models’ accuracy, we performed a five-fold cross validation of both models, keeping the test mean square error as accuracy measurement.

#### Comparing DNase-seq data chromatin accessibility

DNase-seq alignment files were downloaded from the Roadmap Epigenomics data portal at https://egg2.wustl.edu/roadmap/web_portal/processed_data.html ^23^. Only samples corresponding to ESC and Adult anatomical groups were kept. Aligned reads were mapped to promoters, and enhancers. Gene promoters were defined as 5 kb regions surrounding the TSS from Genecode database www.gencodegenes.org/releases/current.html, from these gene promoters we extracted 600 TFs present at HOCOMOCO database https://autosome.ru/hocomoco/ ^41^ to define the TF promoters. Enhancers regions were defined based on Roadmap ChromHMM segmentations data, considering only the distal, non-genic enhancer state from the 15-state model. Reads mapping target regions were aggregated using bedops with the bedmap command^42^. A group mean accessibility score was defined among all ESC and adult samples in every genomic region by calculating mean accessibility across samples of the same group.

#### Implementation

All the methods presented here were implemented using the *R* statistical programming environment www.R-project.org and the igraph package^43^.

## Electronic supplementary material

Supplementary Material

## References

[1] Huang S (2004). Back to the biology in systems biology: What can we learn from biomolecular networks?. Briefings in functional genomics & proteomics.

[2] Barabasi A-L, Oltvai ZN (2004). Network biology: understanding the cell’s functional organization. Nature reviews genetics.

[3] Babu MM, Teichmann SA, Aravind L (2006). Evolutionary dynamics of prokaryotic transcriptional regulatory networks. Journal of molecular biology.

[4] Thiele I (2013). A community-driven global reconstruction of human metabolism. Nature biotechnology.

[5] Li, T. *et al*. A scored human protein-protein interaction network to catalyze genomic interpretation. *Nature methods* (2016). 10.1038/nmeth.4083 PMC5839635 27892958

[6] Newman, M. *Networks: an introduction* (OUP Oxford, 2010).

[7] Costa LdF, Rodrigues FA, Travieso G, Villas Boas PR (2007). Characterization of complex networks: A survey of measurements. Advances in Physics.

[8] Barrat, A., Barthelemy, M. & Vespignani, A. *Dynamical processes on complex networks* (Cambridge University Press, 2008).

[9] Neph S (2012). Circuitry and dynamics of human transcription factor regulatory networks. Cell.

[10] Marbach, D. *et al*. Tissue-specific regulatory circuits reveal variable modular perturbations across complex diseases. *Nature methods* (2016). 10.1038/nmeth.3799 PMC4967716 26950747

[11] Hesselberth JR (2009). Global mapping of protein-DNA interactions *in vivo* by digital genomic footprinting. Nature Methods.

[12] Sullivan AM, Bubb KL, Sandstrom R, Stamatoyannopoulos JA, Queitsch C (2015). Dnase i hypersensitivity mapping, genomic footprinting, and transcription factor networks in plants. Current Plant Biology.

[13] Vierstra J, Stamatoyannopoulos JA (2016). Genomic footprinting. Nature methods.

[14] Stergachis AB (2014). Conservation of trans-acting circuitry during mammalian regulatory evolution. Nature.

[15] Banerji CRS (2013). Cellular network entropy as the energy potential in waddington’s differentiation landscape. Scientific Reports.

[16] Teschendorff AE, Enver T (2017). Single-cell entropy for accurate estimation of differentiation potency from a cell’s transcriptome. Nature Communications.

[17] Kolaczyk, E. D. & Csárdi, G. *Statistical analysis of network data with R*, vol. 65 (Springer, 2014).

[18] Albert R, Jeong H, Barabási A-L (2000). Error and attack tolerance of complex networks. nature.

[19] Schieber TA (2017). Quantification of network structural dissimilarities. Nature Communications.

[20] Zhang Z, Zhang J (2009). A Big World Inside Small-World Networks. PLoS One.

[21] Barabási A-L, Albert R (1999). Emergence of scaling in random networks. Science.

[22] MacArthur BD, Lemischka IR (2013). Statistical mechanics of pluripotency. Cell.

[23] Consortium RE (2015). Integrative analysis of 111 reference human epigenomes. Nature.

[24] Maston GA, Evans SK, Green MR (2006). Transcriptional regulatory elements in the human genome. Annual Review of Genomics and Human Genetics.

[25] Garcia-Ojalvo, J., Arias, A. M. & Martinez Arias, A. Towards a statistical mechanics of cell fate decisions. *Current Opinion in Genetics and Development development* **22**, 619–626, https://www.ncbi.nlm.nih.gov/pubmed/23200114 (2012). 10.1016/j.gde.2012.10.004 23200114

[26] Doyle J (2005). The “robust yet fragile” nature of the internet. Proceedings of the National Academy of Sciences USA.

[27] Turner BM (2008). Open Chromatin and Hypertranscription in Embryonic Stem Cells. Cell Stem Cell.

[28] Meshorer E, Misteli T (2006). Chromatin in pluripotent embryonic stem cells and differentiation. Nature Reviews Molecular Cell Biology.

[29] Spivakov M, Fisher AG (2007). Epigenetic signatures of stem-cell identity. Nature reviews. Genetics.

[30] Marks H (2012). The transcriptional and epigenomic foundations of ground state pluripotency. Cell.

[31] Huang S, Guo Y-P, May G, Enver T (2007). Bifurcation dynamics in lineage-commitment in bipotent progenitor cells. Developmental biology.

[32] Zhou JX, Huang S (2011). Understanding gene circuits at cell-fate branch points for rational cell reprogramming. Trends in Genetics.

[33] Buenrostro JD (2015). Single-cell chromatin accessibility reveals principles of regulatory variation. Nature.

[34] Marr C, Zhou JX, Huang S (2016). Single-cell gene expression profiling and cell state dynamics: collecting data, correlating data points and connecting the dots. Current opinion in biotechnology.

[35] Davila-Velderrain, J., Martinez-Garcia, J. C. & Alvarez-Buylla, E. R. Modeling the epigenetic attractors landscape: toward a post-genomic mechanistic understanding of development. *Frontiers in genetics* **6** (2015). 10.3389/fgene.2015.00160 PMC4407578 25954305

[36] Aldana M (2003). Boolean dynamics of networks with scale-free topology. Physica D: Nonlinear Phenomena.

[37] Valverde, S., Ohse, S., Turalska, M., West, B. J. & Garcia-Ojalvo, J. Structural determinants of criticality in biological networks. *Frontiers in physiology* **6**, 127, http://www.pubmedcentral.nih.gov/articlerender.fcgi?artid=4424853&tool=pmcentrez&rendertype=abstract (2015). 10.3389/fphys.2015.00127 PMC4424853 26005422

[38] Huang, S. Systems biology of stem cells: three useful perspectives to help overcome the paradigm of linear pathways. *Philosophical transactions of the Royal Society of London. Series B, Biological sciences* 2247–2259, 10.1098/rstb.2011.0008. 10.1098/rstb.2011.0008 PMC3130416 21727130

[39] Kaneko, K. Characterization of stem cells and cancer cells on the basis of gene expression profile stability, plasticity, and robustness: Dynamical systems theory of gene expressions under cell-cell interaction explains mutational robustness of differentiated cells. *BioEssays* **33**, 403–413, https://www.ncbi.nlm.nih.gov/pubmed/21538414 (2011). 10.1002/bies.201000153 21538414

[40] Callaway DS, Newman ME, Strogatz SH, Watts DJ (2000). Network robustness and fragility: Percolation on random graphs. Physical review letters.

[41] Kulakovskiy IV (2016). HOCOMOCO: Expansion and enhancement of the collection of transcription factor binding sites models. Nucleic Acids Research.

[42] Neph S (2012). Bedops: high-performance genomic feature operations. Bioinformatics.

[43] Csardi G, Nepusz T (2006). The igraph software package for complex network research. InterJournal, Complex Systems.

