# Supplementary material for: Structural robustness of mammalian transcription factor networks reveals plasticity across development
Source: Sci Rep. 2018 Sep 17;8:13922. doi: 10.1038/s41598-018-32020-1 (PMC6141546; doi:10.1038/s41598-018-32020-1)
Supplement: Supplementary file 1 — Supplementary Material [file 41598_2018_32020_MOESM1_ESM.pdf]

Supplementary Material: Structural robustness of mammalian transcription factor networks reveals plasticity across development

Caldu-Primo JL[1,2], Alvarez-Buylla ER[1,2],Davila-Velderrain J[1,*]

[1]Centro de Ciencias de la Complejidad (C3), Universidad Nacional Autónoma de México, Cd. Universitaria, México, D.F. 04510, México
[2]Instituto de Ecología, Universidad Nacional Autónoma de México, Cd. Universitaria, México, D.F. 04510, México
[*]*JDV. Present address*: MIT Computer Science and Artificial Intelligence Laboratory, Cambridge, Massachusetts, USA. Broad Institute of MIT and Harvard, Cambridge, Massachusetts, USA.
[*]jdavilav@mit.edu

Supplementary Figures

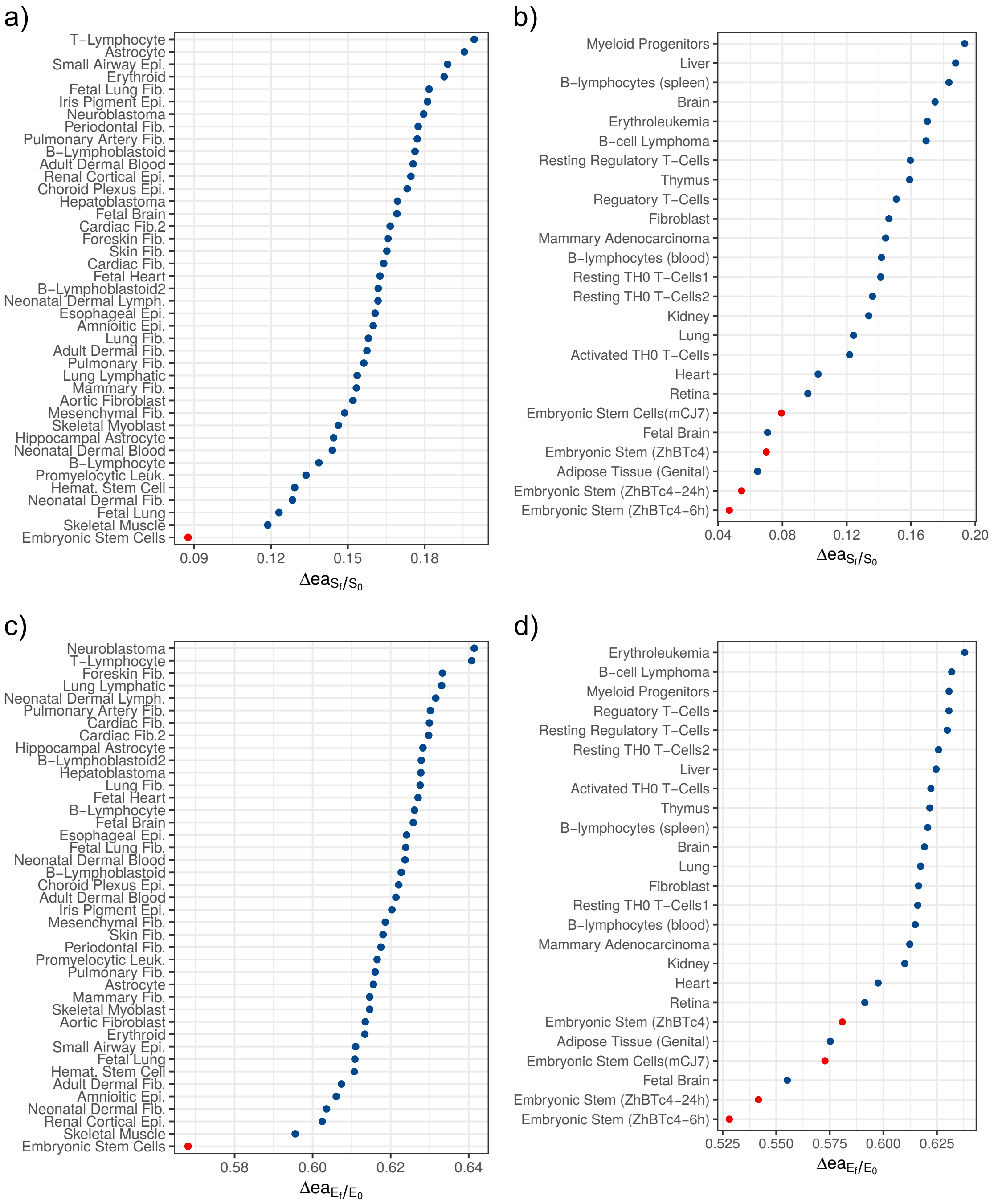

Supplementary Figure 1: Error-attack deviations. Giant component size error-attack deviation for a)human tissues and b) mouse tissues. Efficiency error-attack deviation for a)human tissues and b) mouse tissues. ESCs' are highlighted in red.

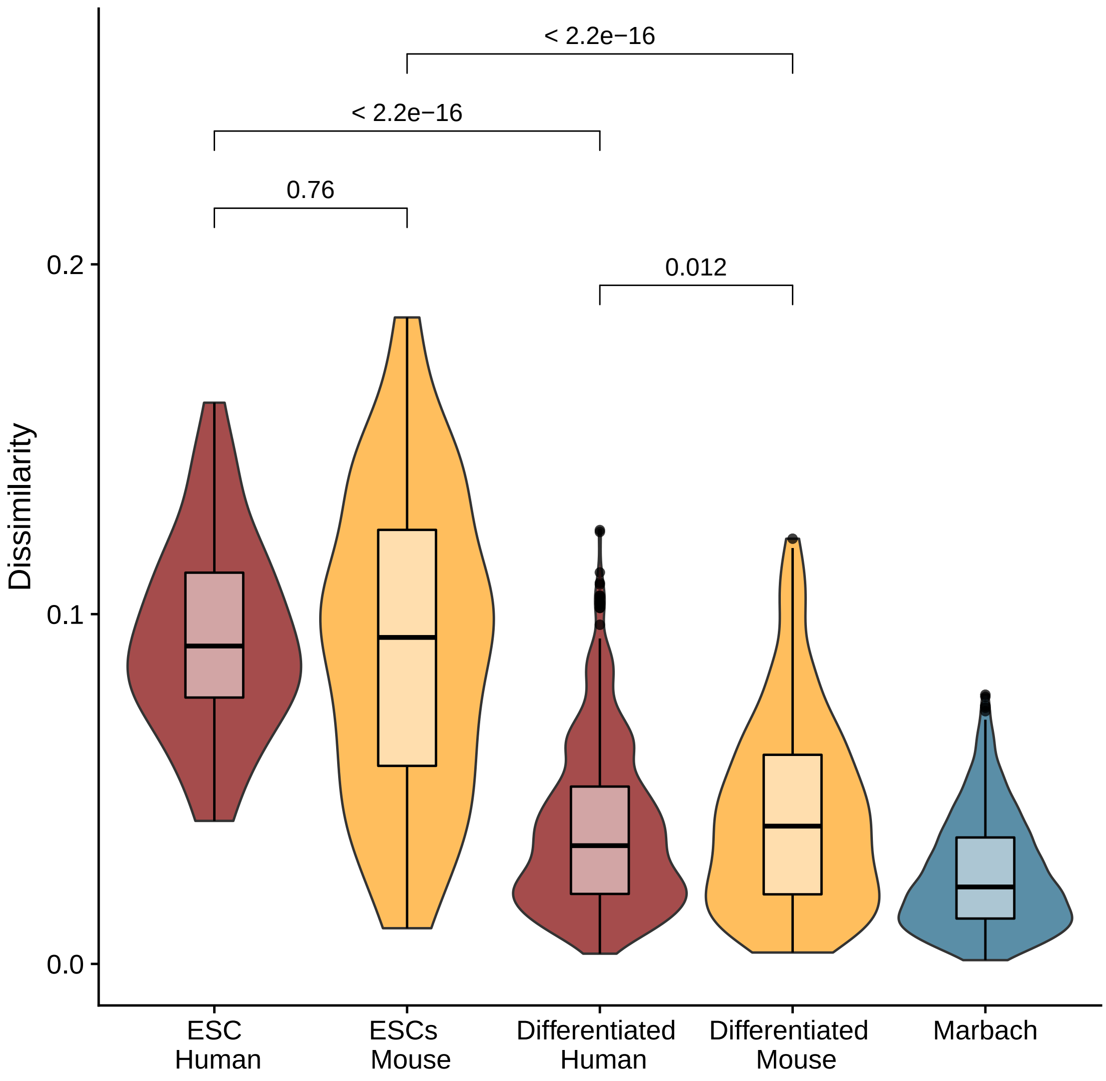

Supplementary Figure 2: Networks dissimilarity distributions between ESC and differentiated cell types and among differentiated cell types. Wilcoxon test p-values comparing means are shown. Mouse differentiated tissues do not include Fetal Brain and Adipose Tissue (Genital) networks. Marbach distribution is the dissimilarity distribution among networks of differentiated cell types published by Marbach et al. (Marbach et al., 2016)

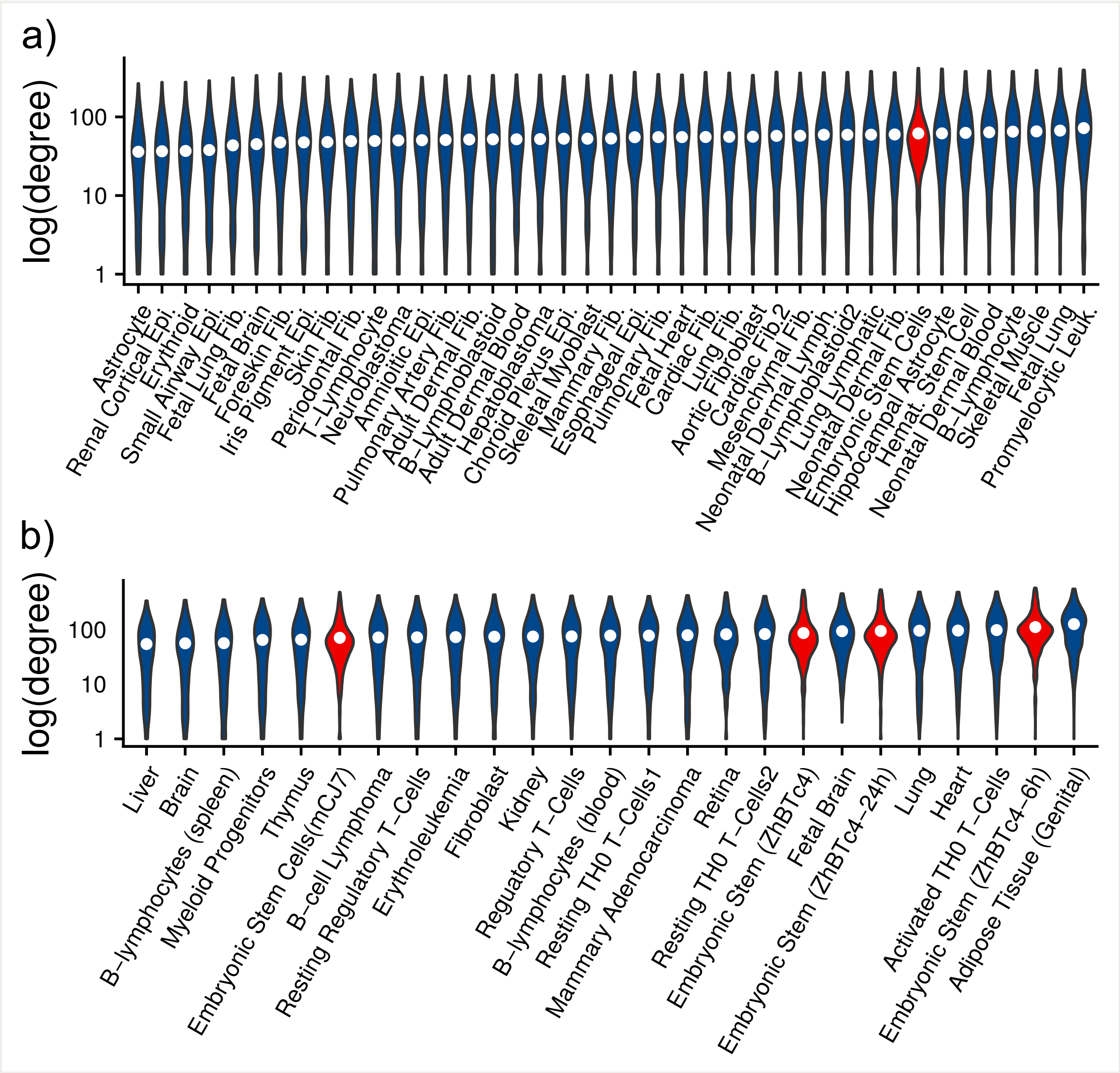

Supplementary Figure 3: Degree distribution of a) human and b) mouse tissues. White dots indicate the value for mean degree, ESCs' distribution is highlighted in red.

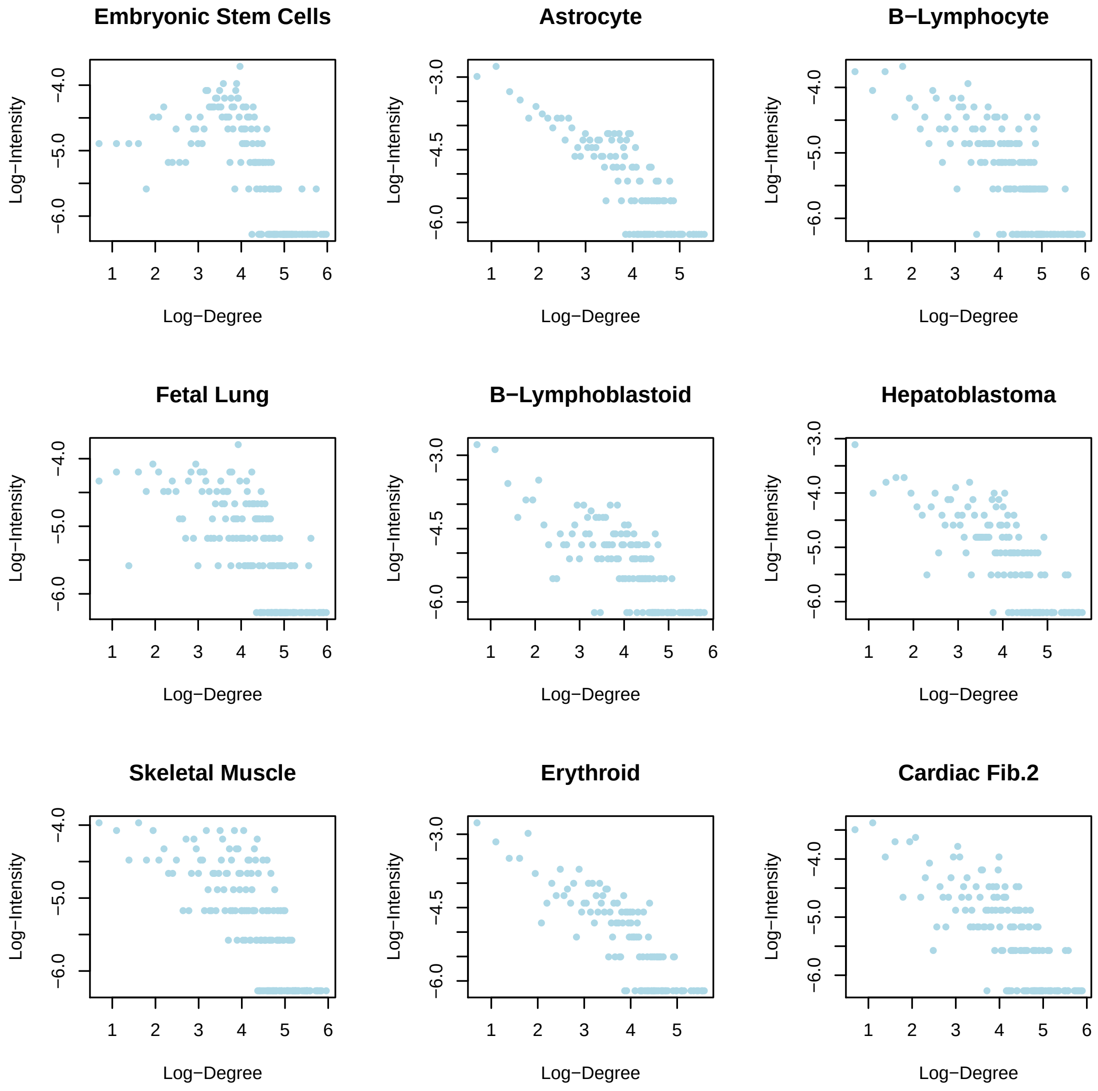

Supplementary Figure 4: Human cell types degree distribution.

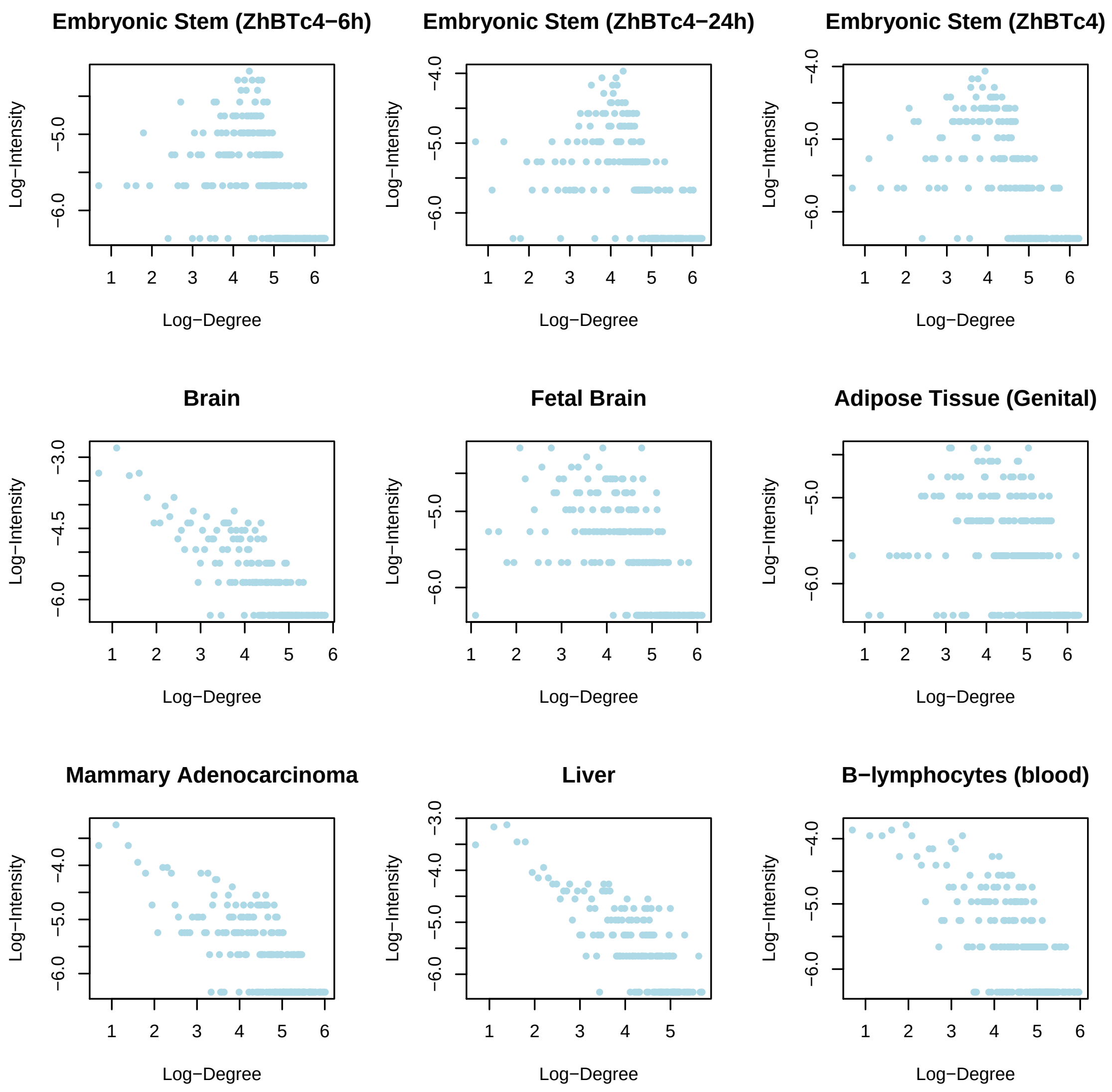

Supplementary Figure 5: Mouse cell types degree distribution.
